# Adjusting for population stratification and relatedness with sequencing data

**DOI:** 10.1186/1753-6561-8-S1-S42

**Published:** 2014-06-17

**Authors:** Yiwei Zhang, Wei Pan

**Affiliations:** 1 Division of Biostatistics, School of Public Health, University of Minnesota, 420 Delaware Street SE, Minneapolis, MN 55455, USA

## Abstract

To avoid inflated type I error and reduced power in genetic association studies, it is necessary to adjust properly for population stratification and known/unknown subject relatedness. It would be interesting to compare the performance of a principal component-based approach with a linear mixed model. Furthermore, with the availability of genome-wide sequencing data, the question of whether it is preferable to use common variants or rare variants for such an adjustment remains largely unknown. In this paper, we use the Genetic Analysis Workshop 18 data to empirically investigate these issues. We consider both a quantitative trait and a binary trait.

## Background

In genetic association studies, population stratification and known or cryptic relatedness are always issues. If not suitably accounted for, these may cause inflated type I errors and reduced power. A popular approach to adjusting for population stratification is to construct principal components (PCs) from some similarity matrix for the samples and to include the PCs as covariates in a regression model [1]. This is referred to as a PC-based approach. However, it is thought that these approaches "do not model family structure or cryptic relatedness" [2]. A more general, and perhaps more powerful, approach is to apply linear mixed models (LMMs) to account for both population stratification and relatedness [3,4]. EMMAX software [5] has facilitated the implementation of LMM by using the identity-by-state (IBS) matrix to capture the complex correlation structure in the samples. As these methods are studied intensely in genome-wise association studies (GWAS), a natural question is how they will perform with sequencing data. It is also of interest to investigate whether common variants (CVs) with minor allele frequencies (MAFs) no less than 0.05, or rare variants (RVs) with 0< MAF < 0.01, should be used to infer the samples' genetic similarities.

In this paper, we compare the PC-based approach with LMM to determine which approach can better control the inflation of type I error arising from correlated samples. The association testing is carried out for a quantitative trait and a binary trait in the Genetic Analysis Workshop 18 (GAW18) family-based sequencing data. For a complete comparison, we construct and consider PCs from different similarity matrices: the sample covariance matrix and the IBS matrix. Finally, we discuss the best choice of variants for constructing the similarity matrix, which has been the subject of several recent studies [6-8].

## Methods

In the PC-based approach, for a given similarity matrix, we obtain its *m *largest eigenvalues *λ_j _*and the corresponding eigenvectors *v_j _*for *j *= 1*, ..., m *and denote $X_{m}=\left(\sqrt{\lambda_{1}}v_{1},\ldots ,\sqrt{\lambda_{m}}v_{m}\right)$. For a quantitative trait, we use a linear regression model *Y *= *β*_0 _+ *X_m_γ *+ *Zζ *+ *gβ *+ *δ *, where *δ ~ N *(0*, σ*^2^*I*). For a binary trait we adopt a logistic model: *Logit*(*E*(*Y *)) = *β*_0 _+ *X_m_γ *+ *Zζ *+ *gβ*.

In the 2 models above, $Y={\left(Y_{1},Y_{2},\ldots ,Y_{n}\right)}^{\prime }$ is the vector of the traits for *n *subjects. $Z={\left(z_{1},\;z_{2},\ldots ,z_{n}\right)}^{\prime }$ is the matrix of covariates, and $g={\left(g_{1},g_{2},\ldots ,g_{n}\right)}^{\prime }$ is the vector of the genotype scores for 1 or more variants to be tested. We denote the method by which PCs are obtained from the IBS matrix as PCA. IBS, and the method by which PCs are obtained from the covariance matrix as PCA.V.

In an LMM, *Y *= *β*_0 _+ *Zζ *+ *gβ *+ *u *+ *δ*, where *Y , Z*, and *g *are defined as above, *u *is the random effect for other polygenic effects and *δ *is the residual error. It is assumed that *δ ~ N*(0, *σ*^2^*I*) and $u={\left(u_{1},u_{2},\ldots ,u_{n}\right)}^{\prime }\sim N\left(0,\sigma_{g}^{2}K\right)$, where *K *is the IBS matrix. We use EMMAX [5] for parameter estimation and inference.

For hypothesis testing, PCA.V and PCA.IBS adopted the Wald test and EMMAX adopted the F-test.

## Results

We used the GAW18 sequencing data containing 959 individuals and 8,348,674 single nucleotide variants (SNVs) across all 11 chromosomes, among which 2,791,923 SNVs were CVs and 3,977,003 were RVs. After pruning by PLINK [9] using a sliding window of size 50, moving step of 5 and *r*^2 ^*<*5%, and filtering out those with missing call *>*0.05, there were 63,157 CVs left. We randomly selected 10,837 CVs from those to construct the similarity matrix. The IBS matrix was obtained by EMMAX, and the covariance matrix was obtained by the R function **cov() **with "use = pairwise.complete.obs" to utilize the maximum number of variants.

We used the measurements of systolic blood pressure (SBP) at time point 1, *SBP*_1_, and the hypertension diagnosis at time point 1, *HTN*_1_. The former is a quantitative trait and the latter is a binary trait. There are 855 samples available. Gender, smoking, and age are the covariates.

### Association test with CVs

We carried out single single-nucleotide polymorphism (SNP) analysis on a set of 6228 CVs randomly selected from all the pruned CVs. Based on the findings of previous GWAS that most of the SNPs were not significantly associated with hypertension, we could assume these 6228 CVs were null SNPs. Because some subjects were from the same families and thus correlated, we expected to observe an inflated type I error if we treated the samples as independent. If the PC-based method or LMM was effective in adjustment, the *p *values should have followed a uniform distribution. This also meant that the proportion of the tests with *p *value *<*0.05 should be close to 0.05 and the inflation factor *λ *close to 1; *λ *is the inflation factor of *p *values estimated by the function **gcontrol2 **in R package **gap**. It is calculated as the ratio of the medians of the observed and expected statistics, respectively.

**Figure 1:**
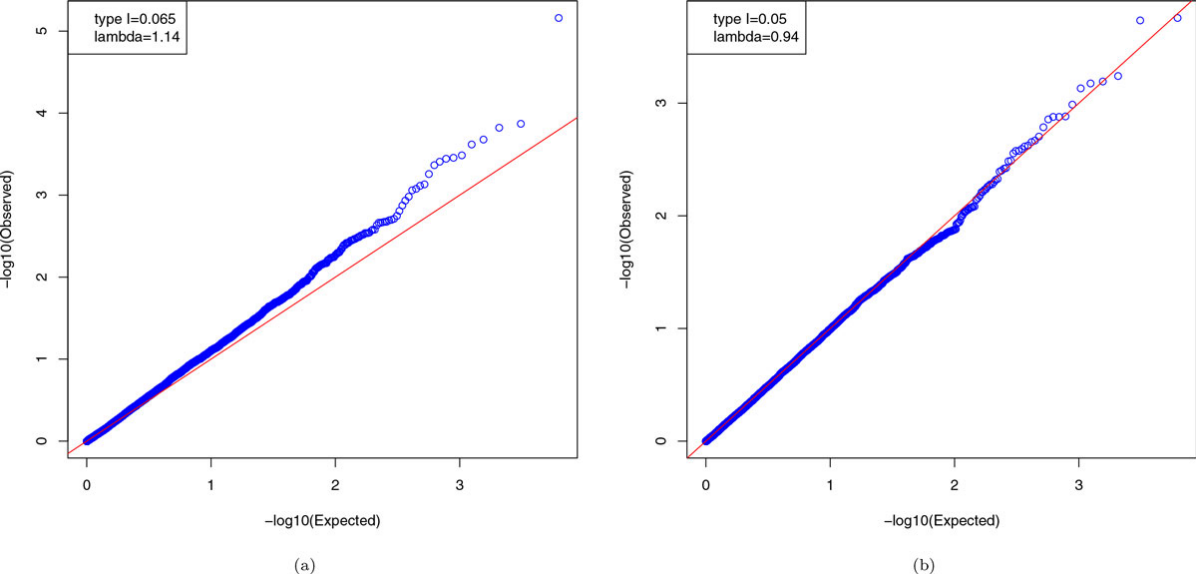
Q-Q plots of *p *values without considering the correlation among samples

Figure 1 shows that, without adjustment, for *SBP*_1 _the observed *p *values deviate from the theoretical uniform distribution (*λ *= 1.14). For *HTN*_1_, the observed *p *values seem to follow the uniform distribution (*λ *= 0.94). This observation might indicate a mild heritability in the GAW18 data set.

**Figure 2:**
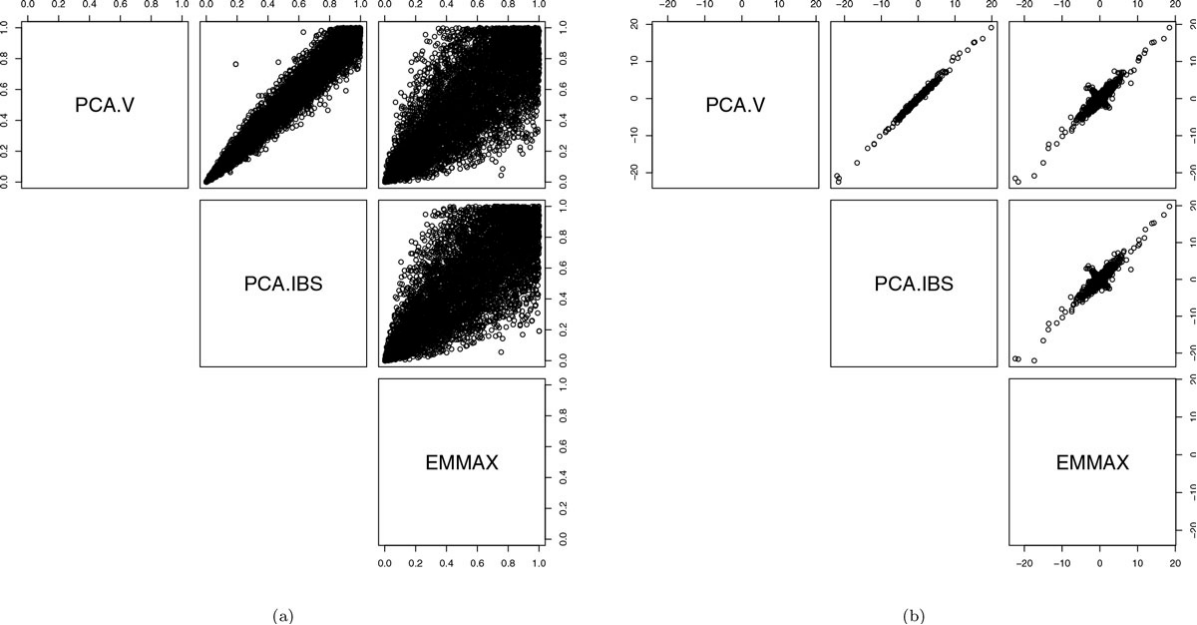
Comparison of PCA.V, PCA.IBS, and EMMAX in testing association between *SBP*_1 _and each of 6228 SNPs

Table 1 shows the quantiles of the association mapping *p *values of *SBP*_1 _with adjustment. We can see the *p *values are almost uniformly distributed. The proportion of the *p *values *<*0.05, estimating the type I error rates, is around 0.05 and of *λ's *around 1. Figure 2 shows some difference between *p *values obtained from the 2 PC-based models and EMMAX. There are also differences in the estimated SNP effects, *β*ˆs. However, both the *p *values and *β*ˆs from the 3 methods are highly correlated.

**Table 1:** Summary statistics of *p *values for *SBP*_1 _by PCA.V, PCA.IBS, and EMMAX. The similarity matrix is based on CVs.

Method	Min.	1st. Qu.	Median	Mean	3rd Qu.	Max.	% (*p *val <0.05)	λ
PCA.V	1.106e-05	0.232	0.486	0.491	0.750	1.000	0.053	1.068
PCA.IBS	5.022e-06	0.235	0.491	0.493	0.749	1.000	0.054	1.041
EMMAX	1.42e-05	0.254	0.516	0.508	0.758	1.000	0.043	0.974

The similarity matrix is based on CVs.

**Figure 3:**
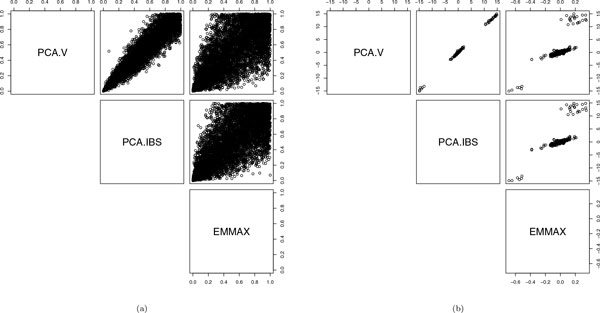
Comparison of PCA.V, PCA.IBS, and EMMAX in testing association between *HTN*_1 _and each of 6228 SNPs

We also apply the methods to the binary trait *H N*_1_. Table 2 shows the *p *values follow a uniform distribution after adjustment. Figure 3 shows that the correlations between the *p *values or *β*ˆs from the 3 methods are weaker than those for *SBP*1. This contrast is partly a result of the logistic link in the PC-based models differing from the identity link in the LMM.

**Table 2:** Summary statistics of *p *values for *HTN*_1 _by PCA.V, PCA.IBS, and EMMAX

Method	Min.	1st. Qu.	Median	Mean	3rd Qu.	Max.	% (*p *val <0.05)	λ
PCA.V	1.457e-04	0.239	0.489	0.494	0.748	1.000	0.055	1.054
PCA.IBS	7.044e-05	0.239	0.492	0.493	0.746	1.000	0.056	1.039
EMMAX	2.831e-04	0.259	0.510	0.507	0.761	1.000	0.048	0.977

The similarity matrix is based on CVs.

### CVs or RVs?

Lastly, we examine which type of variants, CVs or RVs, are more capable of capturing the underlying sample structure. For this purpose, we use PLINK to randomly select 11,103 variants from 1,104,098 pruned RVs to construct the covariance matrix or IBS matrix.

Table 3 shows the results of the association testing, adjusted with PCs of the new similarity matrices based on RVs. PCA.IBS does a satisfactory job of controlling type I errors and *λ*s in testing 6228 CVs for both *SBP*_1 _and *HTN*_1_. EMMAX is a little conservative for *HTN*_1_. Interestingly, we can see a greater distinction between PCA.V and PCA.IBS here than in the previous results, where the similarity matrices were based on CVs. The PCA.IBS is better than the PCA.V at controlling the inflation.

**Table 3:** Results of the association tests by PCA

	% (*p *val <0.05)				λ		
			
	PCA.V	PCA.IBS	EMMAX		PCA.V	PCA.IBS	EMMAX
*SBP*_1_	0.068	0.052	0.052		1.121	1.050	1.054
*HTN*_1_	0.062	0.049	0.049		1.080	1.000	0.980

The similarity matrix is based on RVs.

Originally, the weaker performance of PCA.V based on RVs was thought to be a result of insufficient inclusion of PCs. Following the suggestion of Patterson et al [1], we use the Tracy-Widom test to test how many PCs are necessary to be considered significant [2,8]. The test shows that the top 210 PCs of the covariance matrix all have *p *values smaller than 0.05. However, we fail to obtain reasonable *p *values with 200 PCs included. This might be because the model could not be fitted, given the small sample size.

**Figure 4:**
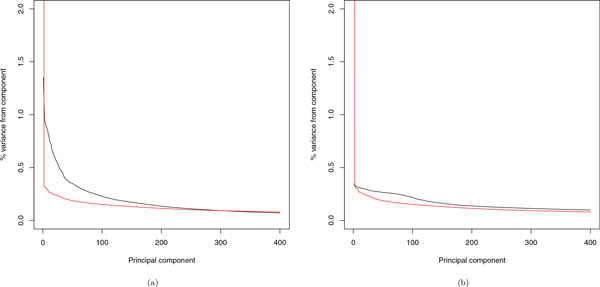
**Scree plots for the top 400 PCs of a similarity matrix based on (a) CVs or (b) RVs**. The black line is for the covariance matrix and the red (gray) line is for the IBS matrix.

Alternatively, we turn to the scree plots (Figure 4) to explain the disparity between the use of CVs and of RVs for a similarity matrix. For the covariance matrix calculated with CVs, there are 458 eigenvalues *>*1, with the top 25 PCs explaining 19.06% of the total variance; for the IBS matrix, there are only 32 eigenvalues *>*1, and the top 25 PCs explain 11.91% of the total variance. For the covariance matrix calculated with RVs, there are 433 eigenvalues *>*1 with the top 25 PCs explaining 7.73% of the total variance; for the IBS matrix, there is only 1 eigenvalue *>*1, with the top 25 PCs explaining 27.02% of the total variance. In short, when using CVs for constructing the similarity matrix, the top 25 PCs of either type can approximate the correlation structure equally well. Although the top 25 PCs of the IBS matrix can still preserve a large proportion of the variation, when using RVs for the similarity matrix, the counterpart of the covariance matrix does a poorer job of approximation.

## Conclusions

In this paper, we address 3 questions: (a) how the PC-based approach and LMM perform in controlling type I error for correlated samples; (b) whether the IBS or covariance matrix should be used to generate PCs; and (c) whether CVs or RVs should be used to construct the similarity matrix. Based on the association testing of 6228 almost uncorrelated CVs from the GAW18 data, we find that PC-based models were capable of taking into account the sample correlations and worked as well as the LMM. This result is different from the claim made in Price et al [2] that PC-based models do not model family structure or cryptic relatedness. When using CVs to construct the similarity matrix, the top few PCs from the IBS matrix and the covariance matrix yield similar results. But when using RVs, the top few PCs from the IBS matrix are slightly better than those from the covariance matrix. LMM implemented by EMMAX is generally as effective as anticipated, although sometimes it can be conservative.

One limitation in our study was that in GAW18 data, there is no serious inflation in type I error for testing *HTN*_1_, even without any adjustment. Although our studies show a positive answer, more studies might be needed to confirm the effectiveness of a PC-based model for testing the binary trait.

## Competing interests

The authors declare that they have no competing interests.

## Authors' contributions

Wei Pan proposed the analysis plan, and Yiwei Zhang provided all statistical analysis and drfted the manuscript. All authors read and approved the final manuscript.
